# Probing the Magnetosheath Boundaries Using Interstellar Boundary Explorer (IBEX) Orbital Encounters

**DOI:** 10.1029/2021JA029278

**Published:** 2021-07-12

**Authors:** S. T. Hart, M. A. Dayeh, D. B. Reisenfeld, P. H. Janzen, D. J. McComas, F. Allegrini, S. A. Fuselier, K. Ogasawara, J. R. Szalay, H. O. Funsten, S. M. Petrinec

**Affiliations:** ^1^ Southwest Research Institute San Antonio TX USA; ^2^ Department of Physics and Astronomy University of Texas at San Antonio San Antonio TX USA; ^3^ Los Alamos National Laboratory Los Alamos NM USA; ^4^ Department of Physics and Astronomy University of Montana Missoula MT USA; ^5^ Department of Astrophysical Sciences Princeton University Princeton NJ USA; ^6^ Lockheed Martin Advanced Technology Center Palo Alto CA USA

**Keywords:** Solar wind, IBEX, magnetopause, bow shock, magnetotail, magnetosheath

## Abstract

Inside the magnetosheath, the IBEX‐Hi energetic neutral atom (ENA) imager measures a distinct background count rate that is more than 10 times the typical heliospheric ENA emissions observed when IBEX is outside the magnetosheath. The source of this enhancement is magnetosheath ions of solar wind (SW) origin that deflect around the Earth's magnetopause (MP), scatter and neutralize from the anti‐sunward part of the IBEX‐Hi sunshade, and continue into the instrument as neutral atoms, behaving indistinguishably from ENAs emitted from distant plasma sources. While this background pollutes observations of outer heliospheric ENAs, it provides a clear signature of IBEX crossings over the magnetospheric boundaries. In this study, we investigate IBEX encounters with the magnetosheath boundaries using ∼8 yr of orbital data, and we determine the MP and bow shock (BS) locations derived from this background signal. We find 280 BS crossings from *X*_GSE_ ∼ 11 R_e_ to *X*_GSE_ ∼ −36 R_e_ and 241 MP crossings from *X*_GSE_ ∼ 6 R_e_ to *X*_GSE_ ∼ −48 R_e_. We compare IBEX BS and MP crossing locations to those from IMP‐8, Geotail, Cluster, Magion‐4, ISEE, and Magnetospheric Multiscale Mission, and we find that IBEX crossing locations overlap with the BS and MP locations inferred from these other data sets. In this paper, we demonstrate how IBEX can be used to identify magnetosheath crossings, and extend boundary observations well past the terminator, thus further constraining future models of magnetosheath boundaries. Furthermore, we use the IBEX data set to show observational evidence of near‐Earth magnetotail squeezing during periods of strong interplanetary magnetic field *B*_y_.

## Key Points

- We demonstrate a technique for producing both Bow Shock and Magnetopause data sets using the Interstellar Boundary Explorer
- We introduce new boundary crossings further tailward than previously used data sets into underexplored magnetosheath regions
- We provide observational evidence of magnetotail squeezing during periods when the interplanetary magnetic field has a strong *y*‐component

## 1 Introduction

The Earth's magnetosphere behaves like a force‐field, creating a barrier between the Earth's atmosphere and the solar wind (SW). The outward magnetic pressure of its magnetosphere balances the inward dynamic pressure of the SW plasma forming the magnetopause (MP). Upstream of the MP, the SW plasma decelerates and deflects around the Earth at the bow shock (BS). The region between the MP and the BS boundaries is the magnetosheath. For the purpose of this paper, the Earth's magnetosphere is assumed to be static (Spreiter et al., 1968). Any change in the magnetosheath boundaries' locations is then a result of the varying upstream SW conditions. The two most notable mechanisms producing boundary motion are compression and expansion caused primarily by changes in SW dynamic pressure, and secondarily by erosion of the Earth's magnetosphere due to magnetic reconnection during periods of southward interplanetary magnetic field (IMF) (Dungey, 1961; Petrinec & Russell, 1993).

Regions in interplanetary space with high density and temperature may produce overwhelming noise in sensitive space plasma instruments, as we will see in this paper, making the magnetosheath location a crucial factor in the development of space‐flight missions looking to avoid its effects. In extreme conditions, the MP can compress to within 6.6 R_e_ sunward and envelop geostationary satellites (Cahill & Winckler, 1999; McComas et al., 1994; Russell, 1976; Shue et al., 1998; Skillman & Sugiura, 1971). A combination of MHD theory, supporting in situ magnetosheath observations, and simultaneous SW measurements over the last 50 yr have enabled a better understanding of the size and shape of the MP (Fairfield, 1971; Formisano et al., 1971, 1979; Petrinec & Russell, 1993, 1996; Shue et al., 1997, 1998; Sibeck et al., 1991) and the BS (Chao et al., 2002; Formisano et al., 1979; Nemecek & Šafránková, 1991; Peredo et al., 1995).

Models of the MP and BS assume their shape to be revolving conic sections about the Earth‐Sun line (Chao et al., 2002; Formisano, 1979; Formisano et al., 1979; Nemecek & Šafránková, 1991; Peredo et al., 1995; Shue et al., 1997, 1998) with some models separating the dayside model from the nightside model (Petrinec & Russell, 1996). The MP models depend only on upstream dynamic pressure and upstream IMF. The BS models additionally depend on upstream Mach number and in some cases the plasma beta (e.g., Chao et al., 2002). The average distances to the MP and BS along the Earth‐Sun line are 11.0 R_e_ and 14.6 R_e_, respectively (Fairfield, 1971). These distances depend primarily on the upstream dynamic pressure. The cross‐sectional widths in the tail region, however, depend strongly on the IMF orientation and Alfvénic Mach number (Haaland et al., 2014, 2019; Merka & Szabo, 2004). Dayside reconnection results in an increase in open magnetic field lines in the Earth's tail region, thus increasing the outward magnetic pressure at the MP and BS flanks causing increased flaring (e.g., Roelof & Sibeck, 1993). In this paper, we demonstrate a novel technique using a distinct background signal observed over nearly a decade by the IBEX‐Hi imager (Funsten et al., 2009) aboard the Interstellar Boundary Explorer (IBEX; McComas et al., 2009) associated with IBEX transit across magnetosheath boundaries, thus adding to the preexisting data sets of in situ crossings.

We use data from the first 352 IBEX orbits, occurring between October, 2008 and January, 2017, and identify 613 magnetosheath crossings. We then compare those with MP and BS modeled locations using upstream SW conditions (Chao et al., 2002; Shue et al., 1998). We further compare the averaged shape of the new MP and BS data sets to those derived from pre‐existing data sets from IMP‐8 and Geotail used in previous models (Jerab et al., 2005; Merka et al., 2003; Nemecek & Šafránková, 1991) and to the BS crossings from Magnetospheric Multiscale Mission (MMS) (Burch et al., 2015). We also discuss the mechanism responsible for creating the background signal in the IBEX‐Hi imager that allows us to perform this analysis. We find that the new MP and BS crossings detected by IBEX are consistent with the preexisting data sets. We identify new magnetosheath crossings further tailward than the data sets mentioned above. These additional crossings allow for further refinement of near tail BS and MP models, and they will assist in the combination of near and far tail crossings (Bennett et al., 1997) to form a more unified magnetosheath model. Finally, we provide observational evidence that a strong IMF *B*_y_ will drape around and compress the magnetotail in the *Z*_GSE_ axis direction, as suggested by Sibeck et al. (1985).

## 2 Data and Instrumentation

**Figure 1:**
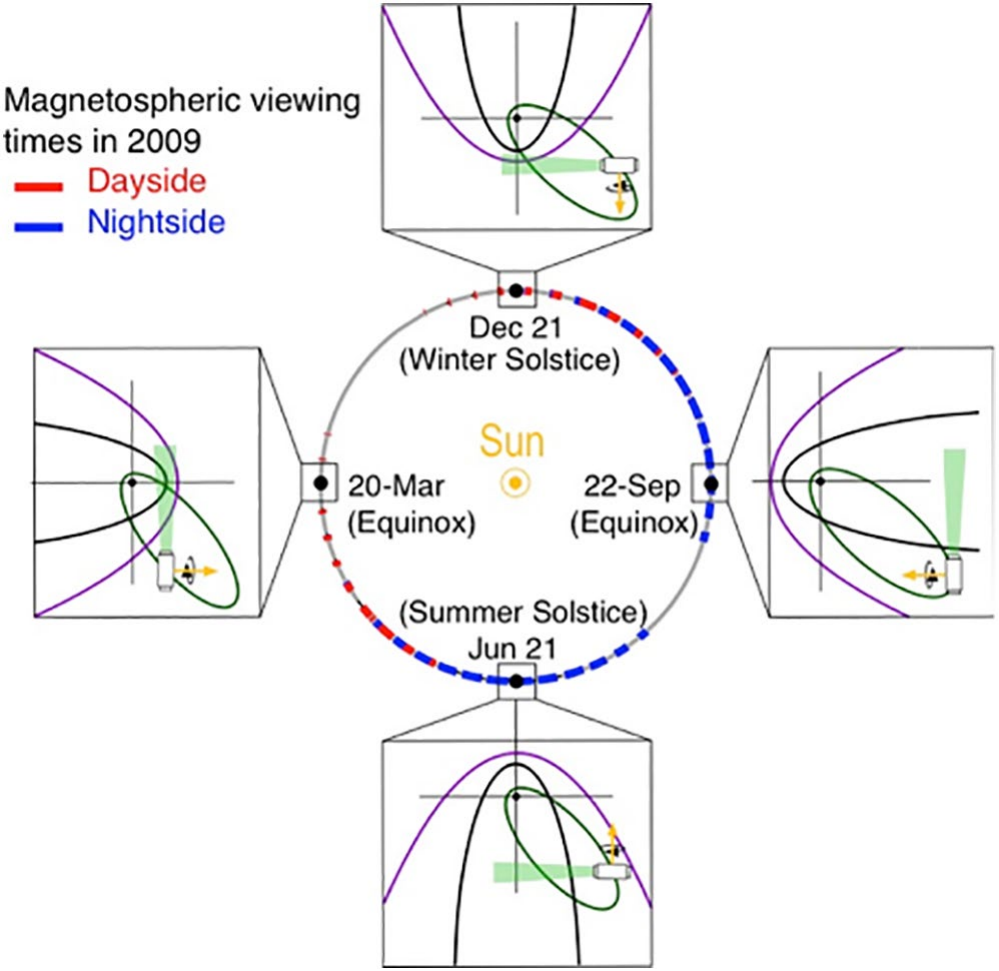
Geometry of IBEX's orbit and magnetosheath viewing in 2009. As it precesses around the Earth over a year, IBEX views and passes through different locations of the magnetosheath covering dayside (red) and night side (blue). After undergoing a perigee raise in 2011 (McComas et al., 2011), IBEX's orbit shifted to ∼10 R_e_ at perigee and ∼50 R_e_ at apogee, not shown above.

IBEX is equipped with two energetic neutral atoms (ENA) imagers, IBEX‐Lo and IBEX‐Hi. The two imagers have overlapping energy ranges allowing for detection of ENAs ranging from 0.01 keV (e.g., interstellar ENAs) to 6 keV (e.g., neutralized pick‐up ions). The IBEX‐Hi ENA imager (Funsten et al., 2009) is a single‐pixel imager with a 6.5° FWHM field of view. IBEX spins with a sunward pointing rotation axis, with each imager viewing a swath of the sky perpendicular to the spin axis. ENAs are measured continuously and also binned onboard into sixty 6° bins over each spin. We use the histogram‐binned data that are accumulated onboard the spacecraft over 48 spins (∼11.5 min) and further accumulated on the ground to 96 spins (∼23 min) representing a swath. Each orbit yields approximately 500, 360° × 6° swaths of ENA data. This study focuses on the second lowest energy pass band (energy step 2), centered at 0.71 keV within which lies the bulk of the deflected SW. IBEX orbits the Earth along a highly eccentric orbital path. Figure 1 shows the annual precession of IBEX's orbit. The orbit of IBEX includes a large part of the magnetosheath during certain seasons due to its high eccentricity. Annually, IBEX spends approximately 18% of its time within the magnetosheath boundaries. In 2011, IBEX was inserted into a stable lunar synchronous orbit, raising the perigee from 2.5 R_e_ to 7.6 R_e_ and moving the orbital period from 7.4 to 9.1 days, i.e., one‐ third of the Moon's orbital period (McComas et al., 2011).

## 3 Methodology

**Figure 2:**
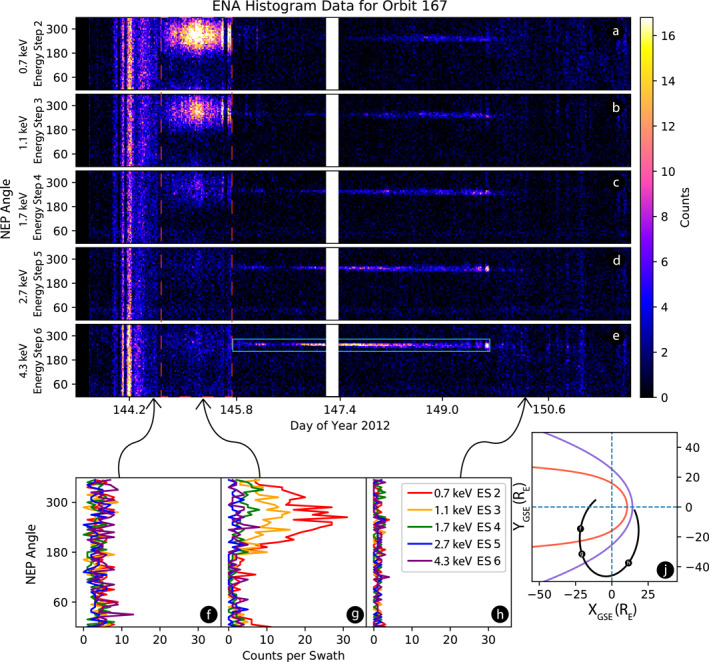
Energy steps 2 (0.7 keV) through 6 (4.3 keV) of the IBEX‐Hi ENA imager during orbit 167 plotted as 2D histograms (top) with the dashed red box indicating the period when IBEX observes the increased signal. Panels (a–e) show the IBEX‐Hi ENA data as a function of north ecliptic pole (NEP) angle and time. A NEP angle of 0°, 90°, 180°, and 270° represents the IBEX‐Hi ENA sensor pointing toward the north ecliptic pole, in the direction of the Earth's motion, the south ecliptic pole, and opposite the direction of the Earth's motion, respectively. Panels (f–h) show histograms of three separate swaths during orbit 167. Panel (j) shows IBEX's orbit and its position during the three sample swaths. The notional BS and MP locations are shown. The count increase in panel (g) is observed when IBEX passes through the Earth's magnetosheath. Panels (f and h) show low count rates that vary little with angle, indicating IBEX is clearly outside the magnetosheath. The Earth's ENA signal is highlighted within the light blue box in panel (e), though this feature exists in all energy pass bands. The white, vertical band at each energy level at the start of DOY 147 is the period separating both arcs of the orbit and indicates the time when IBEX's spin‐axis was repointed to remain sunward facing (McComas et al., 2011).

An example of the IBEX‐Hi ENA data (orbit 167) used in this analysis is shown in Figure 2. The figure shows one orbit of ENA histograms over time, binned every 6° for five consecutive energy pass bands centered on 0.7, 1.1, 1.7, 2.7, and 4.3 keV. The data collection period of orbit 167 began May 24, 2012 and ended on May 31, 2012. Over the course of a single IBEX orbit, the Earth revolves about 8° of its total annual orbit. To prevent light saturation, IBEX repoints its spin‐axis at perigee and apogee (McComas et al., 2011). The white, vertical band at each energy level at the start of day of year (DOY) 147 indicates the apogee of the IBEX orbit. During this time, IBEX's spin‐axis is repointed to remain sunward facing and it does not collect ENA data. Additionally, IBEX does not collect ENA data during the moments leading up to and following perigee to prevent electronic exposure to inner magnetospheric radiation (not shown in Figure 2). The magnetosheath signature is not noticeably affected by this 8° angle range.

As IBEX passes through the magnetosheath, the ENA count rate increases at spin angles opposite to the magnetosheath deflection angle. For instance, dawnward deflected magnetosheath plasma is observed by IBEX in the duskward facing spin angles. Figure 2 highlights this interaction. IBEX passed through the magnetosheath during DOY 144.68–145.79, shown in the dashed red box. The count rates of a single swath during this pass are shown in panel (g). Throughout this time, energy steps 2, 3, and 4 observed increased count rates between spin angle 180° and 360° of over 10 times the typical measured count rates. Energy steps 5 and 6 show little to no increase in the count rate within the magnetosheath, because they are above the typical energy of the core SW. Outside the magnetosheath, IBEX observes ENA signal from the heliosphere like that of panel (h). Inside the magnetosheath, the count rate is most enhanced in the second energy step (0.7 keV) and is therefore the energy step used for our analysis. IBEX's position during orbit 167 is shown in the bottom right of Figure 2 along with models of the BS and MP positions (Chao et al., 2002 for BS; Shue et al., 1998 for MP) using upstream averaged SW conditions from the OMNIWeb database. As IBEX orbits counterclockwise, it passes points (f), (g), and (h). At these locations, IBEX observes the swaths shown in panels (f–h) respectively. Between swaths (f) and (g), IBEX crosses the MP and enters the magnetosheath on the dawnside. The ENA count rates increase in the duskward facing spin angles, evident in panel (g). Later, IBEX crosses the BS and leaves the magnetosheath, and the count rates drop sharply.

Highly anisotropic signatures are not unique to the background signal created in the magnetosheath. For instance, IBEX detects remote ENAs produced within the Earth's magnetosphere (Dayeh, 2015; Fuselier et al., 2010) and magnetosheath (Dayeh et al., 2020; Ogasawara et al., 2013), and IBEX detects ENAs that were previously neutralized after a collision with the lunar surface (Allegrini et al., 2013; Funsten et al., 2013; McComas, 2010). The Earth's ENA signature appears in Figure 2 (blue box) during DOY 146 through 148 along spin angle 270° most noticeably in the higher energy steps. We distinguish the in situ magnetosheath background signal from the other remote sources by its width in the sky, as well as the expected direction of the largest signal increase based on IBEX's location.

**Figure 3:**
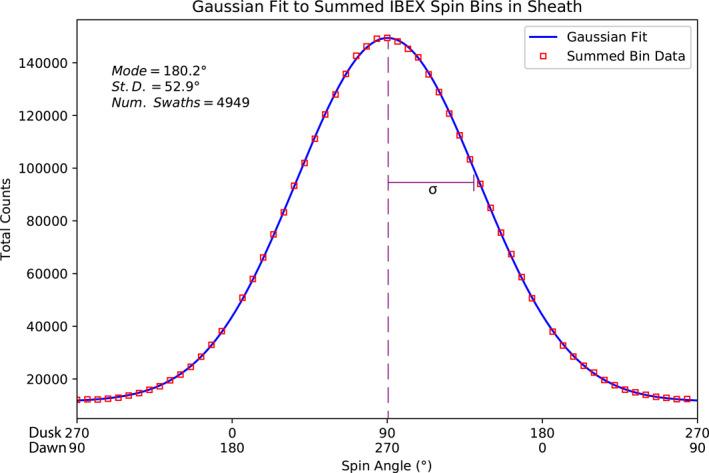
Summation of 4,949 swaths when IBEX is within the magnetosheath boundaries. The summation is fit to a Gaussian distribution successfully, allowing us to fit a Gaussian to individual swaths for further analysis.

We sum the angle bins of the swaths with the largest count rate increase where IBEX is believed to be within the magnetosheath boundaries, such as the swath from Figure 2g. Figure 3 shows the response of this accumulation, which is well‐described by a Gaussian function. To account for the discontinuity of data between angles 360° and 0° within the same swath, we shift the data by ±90° (dusk or dawn) thus ensuring that an optimal Gaussian fit is performed. The Gaussian response shown in Figure 3 comprises 4,949 swaths and clearly illustrates that a Gaussian distribution describes this signal well. The cumulative response shows that the background signal is centered around spin angle 90° (dusk observations) or 270° (dawn observations), with a standard deviation of 52.9°. We performed this fit on energy steps 2 through 4 and found that the reduced chi‐square value of the fit was nearest to one for energy step 2, further confirming our choice to use it for analysis.

We now require each swath to have Gaussian‐like characteristics in order for it to be considered a magnetosheath crossing observation. Three constraints on the characteristics of the fitted Gaussian are used to determine the crossings. These constraints were determined heuristically from the analysis. First, each Gaussian's amplitude parameter must have a peak‐to‐variance ratio of at least 7.5 (decreasing tailward to match decreasing background signal). A large peak‐to‐variance ratio is achieved through high count rates with little deviation in the Gaussian structure. Second, the width of the Gaussian fit is confined to be near 52.9° (calculated as σ from Figure 3). Last, the reduced chi‐square of the fit must be between 0.4 and 3.0. If all three conditions are met, the swath is considered a potential magnetosheath observation and is denoted as a positive instance. Otherwise, it is assumed the magnetosheath signature is not observed and the swath is denoted as a negative instance.

**Figure 4:**
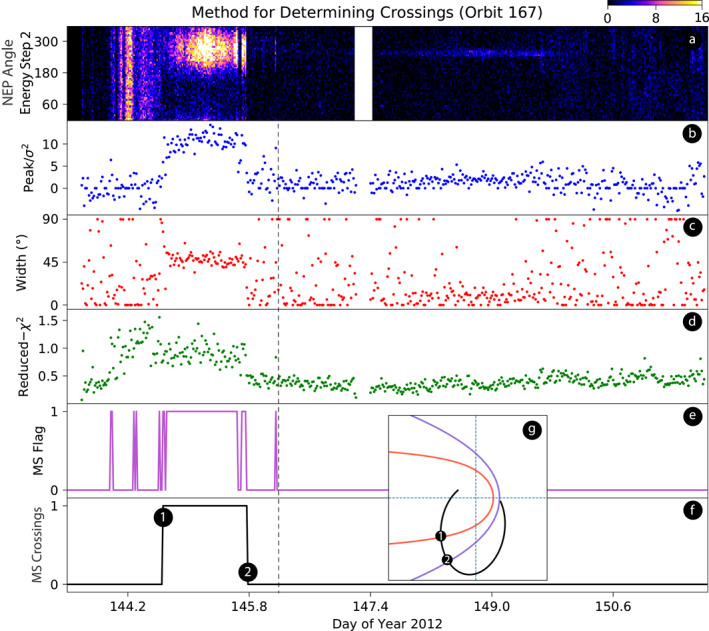
Methodology for determining IBEX's magnetosheath crossing times. Figure 4a shows ENA data from IBEX‐Hi energy step 2 (0.7 keV). Figures 4b–4d show characteristics of the Gaussian fit performed on each swath. Swaths with high peak‐to‐variance ratios, Gaussian widths near 52.9°, and reduced chi‐square values near unity satisfy the conditions necessary to be considered a potential magnetosheath observation. If all the conditions are met, the magnetosheath (MS) flag in Figure 4e equals one, otherwise it equals zero. Refining the flags by removing isolated positives and smoothing out the jagged edges yields the final result shown in Figure 4f. During orbit 167, IBEX entered the magnetosheath, crossing the MP at DOY 144.68, and exited across the BS at DOY 145.79. Figure 4g shows IBEX's location at the time of the boundary crossings. The black dots show the entrance (1) and the exit (2) locations obtained using this methodology.

Figure 4 illustrates the heuristic method developed to identify a boundary crossing. Figure 4a shows the ENA data from energy step 2. Figure 4b is the first of the three criteria, showing the peak‐to‐variance ratio of the Gaussian fit for each swath; Figure 4c shows the corresponding standard deviation of the data; and Figure 4d shows the reduced chi‐square values of the fits. Regarding Figure 4d, when IBEX is outside the magnetosheath the Gaussian fitter is over‐fitting the background heliospheric ENAs, and as a result the reduced chi‐square values for these swaths are significantly less than one. Figure 4e flags the magnetosheath detections for each swath as 0 (negative; no magnetosheath) and 1 (positive; magnetosheath detected). For IBEX to cross the magnetosheath, we set a conservative condition to have 8 positive instances within 12 consecutive swaths (75% positive instances). For IBEX to exit the MS, we require 8 negative instances within 12 consecutive swaths (75% negative instances). This corresponds to a time scale (∼3 h) that is significantly larger than a typical crossing time (minutes). Nonetheless, this conservative requirement enables only the most certain crossings. As a result, isolated positives are not considered magnetosheath observations. If this condition is met, the first positive/negative swath in the 12 consecutive swaths is counted as the entrance/exit. In this case, every swath from DOY 146.4 onward is a negative instance (right of the vertical dashed line. Prior to that, there are 71 positive instances, five of which are isolated. The rest of the positive instances are continuous or clumped between DOY 144.5 and 146.

The final result of the crossing identification is shown in Figure 4f. Crossings are flagged “zero” outside the magnetosheath and “one” inside the magnetosheath. Any shift from zero to one or vice versa indicates a crossing. For orbit 167, the entrance and exit occur at DOY 144.68 and 145.79, respectively meaning IBEX's pass lasted approximately 26.6 h. IBEX's orbit is shown in Figure 4g along with the MP and BS models. The black circles (noted as 1 and 2) along IBEX's orbit indicate IBEX's position at the time of the crossings. The magnetosheath boundary models shown here use the 23 min averaged SW conditions at the time of the respective crossing and line up well with crossing locations obtained using our methodology.

## 4 Results and Discussion

**Figure 5:**
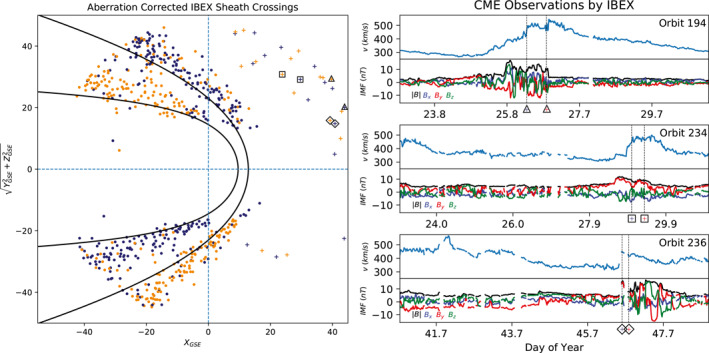
(a) IBEX's location during the 613 magnetosheath boundary crossings. Entrances are shown in navy, and exits are shown in orange. The crossings have been corrected for the Earth's orbit around the Sun and the solar wind's incident angle to maintain axial symmetry. The hollow shapes upstream of the magnetosheath highlight a sample of IBEX detections of ICMEs during different orbits. (b) A sample of three ICME observations by IBEX. The top panel of each orbit is the solar wind speed, and the bottom panel shows the IMF components. The dashed vertical lines indicate the region where the IBEX‐Hi ENA signal was flagged as a magnetosheath observation. The shapes below the dashed lines match the shapes in Figure 5a, showing where IBEX was located during the ICME pass.

Applying the magnetosheath crossing methodology described above to 352 IBEX orbits yields 613 boundary crossings. Each boundary crossing is adjusted to correct for the SW aberration. The result is shown in Figure 5a. Entrances into the magnetosheath are shown as navy dots, and exits out of the magnetosheath are shown as orange dots. Cylindrical symmetry of the BS and MP about the *x*‐axis is assumed, thus we rotate the crossings' *Z*‐components into the *XY*‐plane. The BS and MP boundaries during average SW conditions are shown. The average SW values used for this analysis are 〈p〉 = 3.1 nPa for dynamic pressure and 〈*M*_MS_〉 = 5.33 for magnetosonic Mach number as listed in Peredo et al. (1995). IBEX was launched during the prolonged minimum of solar cycle 24 (Jiang et al., 2015; McComas, Angold, et al., 2013). The dynamic pressure values during this period were consistently below average. The average dynamic pressure at the time of IBEX's boundary crossings is 〈p〉 = 1.9 ± 1.5 nPa, while the average dynamic pressure listed in Peredo et al. (1995) is 〈p〉 = 3.1 nPa and 〈p〉 = 3.2 nPa as calculated from Nemecek and Šafránková (1991). As a result, the majority of IBEX's crossings lie outside the BS and MP models from Figure 5a.

All of the crossing points located far upstream of the magnetosheath (top right and bottom right of Figure 5a, shown as plus signs) are not magnetosheath observations, and are in fact IBEX observations of shock‐associated transient events such as interplanetary coronal mass ejections (ICMEs; Dayeh, 2015; Dayeh et al., 2015; Jian et al., 2006) and corotating interaction regions (Smith & Wolfe, 1976) which generate a similar type of background in IBEX‐Hi. Figure 5b shows three examples of ICME detections using the OMNIWeb 1 min data of the SW speed and the IMF. OMNIWeb SW plasma data is propagated to the BS nose position. Further propagation is not needed for magnetosheath and shock‐associated observations as the maximum time difference is significantly less than the IBEX bin resolution of 23 min. An ICME is preceded by an interplanetary shock that is characterized by a sudden jump in the SW speed and the IMF strength (Burlaga et al., 1982). For each event shown, there are two dashed vertical lines. These dashed lines indicate the beginning and end of a large increase in background signal flagged as a magnetosheath observation based on the criteria from the Methodology section. The shapes under the dashed lines in Figure 5b are plotted in Figure 5a showing IBEX's location at the time of the event.

**Figure 6:**
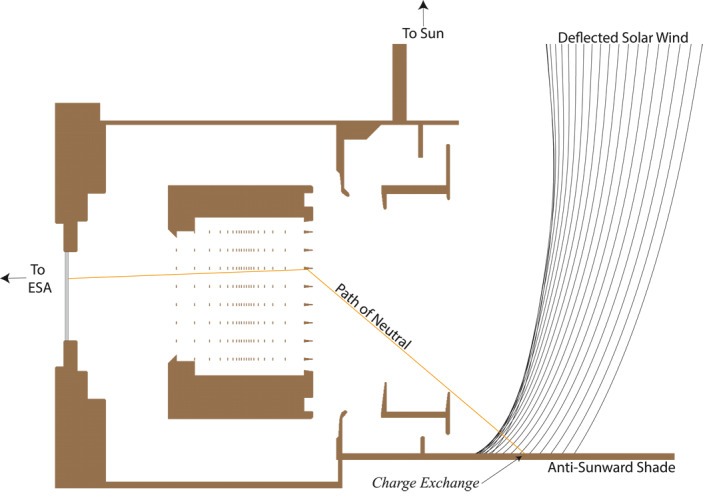
Schematic of the IBEX‐Hi ENA entrance aperture geometry during the signal production process. Deflected solar wind neutralizes with the anti‐sunward shade of IBEX‐Hi and scatters into the collimator. A series of collisions within the collimator redirects the newly produced ENA toward the carbon foil where it becomes indistinguishable from a heliospheric ENA. This process is highly unlikely, but the flux of the deflected solar wind is large enough such that only 1 in 10^10^ solar wind protons must succeed to produce the observed signal in Figure 2.

The prevailing explanation for the signal production process is shown in Figure 6. Figure 6 shows a cross‐section of the IBEX‐Hi entrance aperture zoomed in on the anti‐sunward portion of the instrument. Deflected SW plasma (from the magnetosheath, ICME, shock, etc.) scatters off the anti‐sunward sunshade of the IBEX‐Hi imager and is neutralized during the collision. The newly produced ENA can traverse the collimator unaffected by the internal potentials. The exact number of collisions within the collimator is unknown, yet we do not believe a set number of collisions is required. If a collision scatters the neutral toward the carbon foil, it becomes indistinguishable from a heliospheric ENA. IBEX observations of passing shocks, within which SW deflection is common (Owens & Cargill, 2004), further supports this mechanism in which hot SW can enter the IBEX‐Hi imager and scatter from interior sources. Both the magnetosheath and shock‐associated transients are hot plasmas with significant $v_{⊥}$ components (perpendicular to the Earth‐Sun line) where these observations are most likely to arise.

The shape and location of the BS and MP can be modeled solely using upstream SW conditions. Spreiter et al. (1968) provide an equation to obtain the distance to the MP and BS along the Earth‐Sun line using the SW speed, density, and Mach number. The equation is as follows:

(1) $$D+\Delta =\frac{C}{\sqrt[{6}]{NV^{2}}}\left(1+1.1\left(\frac{(\gamma -1)M^{2}+2}{(\gamma +1)M^{2}}\right)\right)$$

where: D is the distance to the MP nose (in Earth radii);

Δ is the magnetosheath thickness along the Earth‐Sun line (in Earth radii);
N is the SW number density (in cm^−3^);
V is the SW velocity (in km s^−1^);
M is the interplanetary Mach number;

C is a constant, usually taken to be 100 (Nemecek & Šafránková, 1991) and γ is assumed to be 5/3. The first term on the right‐hand side describes the distance to the MP nose, and the second term on the right‐hand side adds the magnetosheath thickness to attain the distance to the BS nose. To account for the variation in SW conditions, each crossing location is normalized radially based on the SW conditions at the time of the crossing and the average SW conditions. We normalize each crossing location using Equation 1 such that the normalized MP locations are:

(2) $$R_{\text{MP,norm}}=R_{\text{MP,obs}}\frac{D_{\text{avg}}}{D_{\text{obs}}}$$

and the normalized BS locations are:

(3) $$R_{\text{BS,norm}}=R_{\text{BS,obs}}\frac{{\left(D+\Delta \right)}_{\text{avg}}}{{\left(D+\Delta \right)}_{\text{obs}}}$$

where $R_{\text{MP,norm}}$ and $R_{\text{BS,norm}}$ are the normalized MP and BS locations for average SW conditions, $R_{\text{MP,obs}}$ and $R_{\text{BS,obs}}$ are the IBEX observed MP and BS locations. D is defined from Equation 1 as:

(4) $$D=\frac{C}{\sqrt[{6}]{NV^{2}}}$$

**Figure 7:**
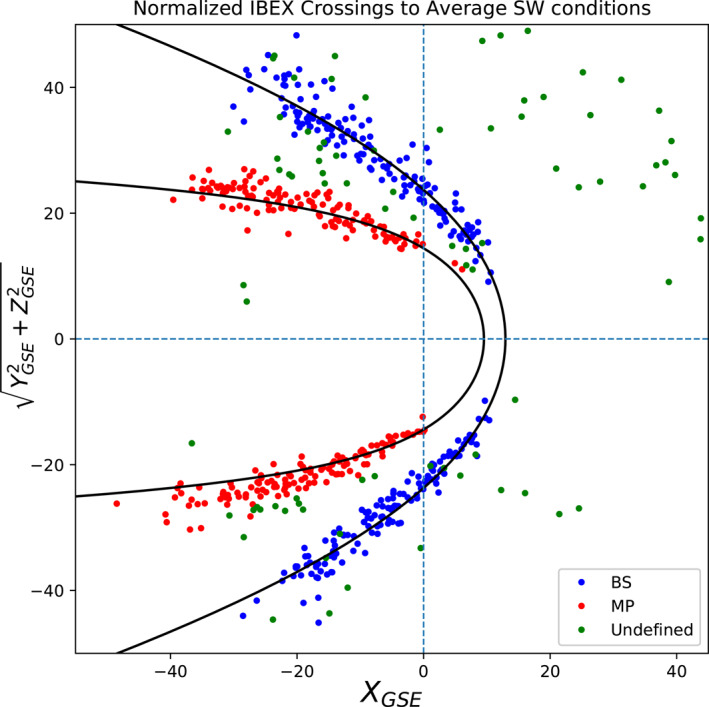
After determining to which boundary each point is attributed, we applied Equation 2 to MP crossings and Equation 3 to BS crossings to normalize the crossing locations to average solar wind conditions, obtaining the figure above. The BS and MP crossings are shown in blue and red, respectively. The crossings colored green could not be associated with a specific boundary with a greater than 67% certainty.

We use 23 min averaged SW conditions from the OMNIWeb database to find the SW conditions at the time of the observed IBEX crossings, and we also use 〈*N*〉 = 7.76 cm^−3^, 〈*V*〉 = 454 km s^−1^, and 〈*M*_MS_〉 = 5.33 as the average SW conditions (Peredo et al., 1995). Equations 2 and 3 assume that the dynamic pressure and magnetosonic Mach number only control the size of the boundaries, not the shape. Applying Equations 2 or 3 to each observed crossing yields the normalized crossings, shown in Figure 7. We use the magnetosonic Mach number for the Mach number in Equation 1, as it considers the most SW properties (Nemecek & Šafránková, 1991). A clear separation between the BS (blue) and MP (red) arises from this technique. To determine the boundary type, we find the distance between each unnormalized crossing and both modeled boundaries at the time of the crossing, and take the ratio of the distances. Crossings not located near either of the boundaries could not be assigned one. In total, 280 crossings are determined to be BS crossings, 241 are MP crossings, and the remaining 92 crossings are too uncertain for boundary assignment.

**Figure 8:**
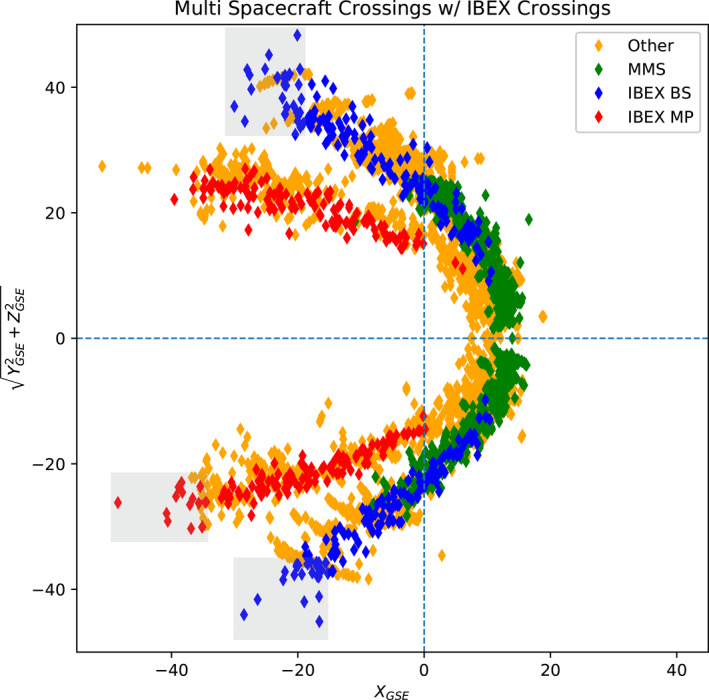
Normalized IBEX crossings (blue: BS, red: MP) from Figure 7 compared with previously used data sets from IMP‐8, Geotail, Cluster, Magion‐4, and ISEE (orange) and new bow shock crossings from MMS (green). Importantly, the IBEX crossings obtained using the methodology explained in this paper agree with previous data sets. Highlighted in gray are IBEX boundary crossings that were observed in previously unexplored magnetosheath regions. These crossings are useful for modeling the night‐side bow shock and MP.

Finally, we compare IBEX crossings along the MP and BS curvatures to those of other spacecraft. We plot the well‐defined IBEX crossings with popular BS data sets from IMP‐8, Geotail, Cluster, and Magion‐4 (1995–2002), and MP data sets from IMP‐8, ISEE, and Geotail (1973–1992) obtained from OMNIWeb. We include new BS crossings from the MMS. The results are shown in Figure 8. Our normalized IBEX crossings (blue: BS, red: MP) are plotted with the unnormalized boundaries from the other spacecraft. Figure 8 shows that the IBEX crossings, having significantly smaller than average dynamic pressures, look consistent with the unnormalized boundary crossings of the other spacecraft once the IBEX crossings are normalized to historically average SW conditions (i.e., the typical SW conditions at the time of the older spacecraft). Because the orbit of IBEX is such that its apogee extends to ∼50 Earth radii, this data set provides crossings in previously unexplored regions of the night‐side magnetosheath, highlighted in gray.

## 5 Magnetotail Squeezing

**Figure 9:**
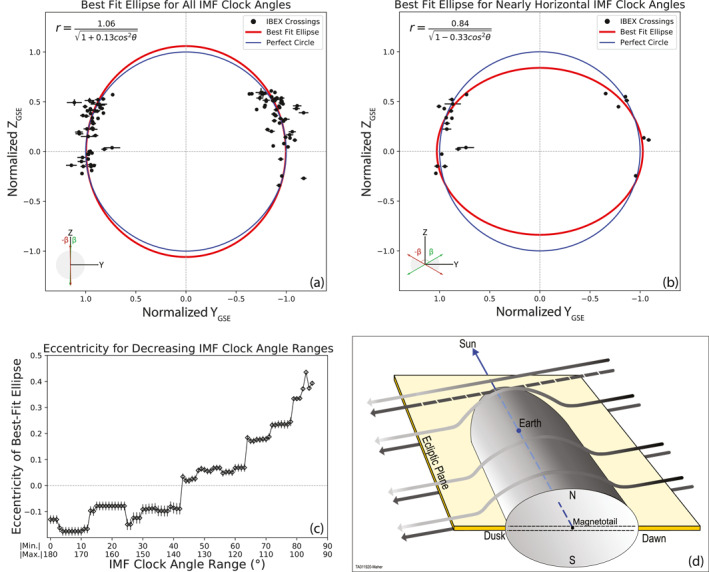
Observational evidence of magnetotail compression along the *Z*_GSE_ during periods of strong IMF *B*_y_. (a) All IBEX MP crossings with *X*_GSE_ < −24 R_e_ and the corresponding best‐fit ellipse. (b) IBEX MP crossings whose corresponding IMF clock angle is 80° < |β| < 100° fitted by an ellipse whose semimajor axis is along the *Y*_GSE_ axis. (c) The eccentricity of the ellipse as a function of decreasing IMF clock angle range. As we restrict the IMF clock angle toward the ecliptic plane, the eccentricity of the best‐fit ellipse increases. The dashed horizontal line at eccentricity = 0 indicates a circular fit. Fits below the dashed line exhibit MP shapes like the fit in (a), and fits above the line exhibit MP shapes like that in (b). (d) An illustration of the phenomenon, adapted from McComas, Dayeh, et al. (2013). The IMF drapes around and compresses the northern and southern‐most regions of the magnetotail.

Much less is known about the high latitude magnetosphere than is known about its equatorial region. Recent studies of the high latitude magnetosphere and magnetosheath were accomplished through the Cluster mission (Kruparova et al., 2019; Phan et al., 2003; Vontrat‐Reberac et al., 2002) with an apogee of 19.6 R_e_. Beyond the orbit of Cluster, plasma and magnetic field measurements of the high latitude magnetotail region are scarce and thus the physics in this regime is not entirely understood. Sibeck et al. (1985) suggested that when the IMF has a strong *B*_y_ paired with a weak *B*_z_, the IMF drapes around and compresses the magnetotail such that the distance to the boundary along the *Z*_GSE_ axis is reduced compared to the *Y*_GSE_ axis. This physical effect was also suggested to occur at the downwind heliospheric tail, supported by heliospheric ENA observations (McComas, Dayeh, et al., 2013). An illustration of this phenomenon is shown in Figure 9d. Using the IBEX MP crossing data set, we find the 23 min averaged IMF clock angle (defined as the angle between the *Z* and *Y B*_IMF_ components) at the time of the IBEX crossings, and show that IBEX crossings point to an elliptical magnetotail at high clock angle values (i.e., *B*_IMF_ parallel to the ecliptic plane).

IBEX has an orbital inclination (>10°) with respect to the Earth's equatorial plane allowing for MP crossings ranging from *Z*_GSE_ = −8.3 R_e_ to *Z*_GSE_ = 16.7 R_e_ for the data set used here. Following Sibeck et al. (1985), IBEX boundary crossings during periods of strong IMF *B*_y_ should occur at smaller |*Z*_GSE_| values than the MP model would predict using the upstream SW conditions (Shue et al., 1998). We first normalize the IBEX crossings by dividing IBEX's (*Y*_GSE_^2^ + *Z*_GSE_^2^)^1/2^ location at each crossing by the Shue et al. (1998) MP model's predicted (*Y*_GSE_^2^ + *Z*_GSE_^2^)^1/2^ distance at the same *X*_GSE_ location. This process ensures that further analysis is independent of the tailward distance of the crossing. We divide again by the average of the normalized values to center them around 1.0, thus allowing the crossing data to be fit easily by an ellipse. We then fit a subset of these points to an ellipse using the equation:

(5) $$r=\frac{d_{z}}{\sqrt{1-ecos^{2}\left(\theta \right)}}$$

where $d_{z}$ is the distance to the ellipse along the *Z*_GSE_ axis, r is the distance to the ellipse for any given angle, θ, and e is a modified eccentricity of the ellipse calculated by:

(6) $$e\;=\;1\;-\;{\left(\frac{d_{z}}{d_{y}}\right)}^{2},\;e:\left(-\infty ,\;1\right)$$

where $d_{y}$ is the distance to the ellipse along the *Y*_GSE_ axis. The relation between the modified eccentricity and the true eccentricity is such that:

(7) $$d_{z}<d_{y}\to e_{\text{true}}=\sqrt{e}\;\;d_{z}>d_{y}\to e_{\text{true}}=\;\sqrt{\frac{e}{e-1}}$$

where $e_{\text{true}}$ is the true eccentricity of the ellipse. A negative eccentricity corresponds to a semimajor axis along the *Z*_GSE_ axis, a positive eccentricity corresponds to a semiminor axis in the same direction, and an eccentricity of zero is a perfect circle. This particular equation keeps the center of the ellipse at the origin, which in this case, is in the magnetotail facing Sunward. Figure 9a shows the best‐fit ellipse for all IBEX MP crossings with *X*_GSE_ < −24 R_e_.

The equation of the best‐fit ellipse for all of the MP crossings is shown in the top left of Figure 9a, and the best‐fit ellipse is shown in red, along with a perfect circle in blue. The selected IBEX MP crossings are in black. The best‐fit ellipse nearly resembles a perfect circle, having an eccentricity of e  = −0.130 ± 0.016: a slight expansion along the *Z*_GSE_ axis. The uncertainty values of the crossings are calculated based on the maximum and minimum SW dynamic pressure values from the OMNIWeb 1 min data set during the 23 min swath interval. Crossings with 12 or less pressure values during their respective 23 min interval are removed from the data used in the fit. Figure 9b shows the best‐fit ellipse of only the MP crossings that occurred during periods when the IMF clock angle was 80° < |β| < 100°. The eccentricity of the best‐fit ellipse for these selected crossings is e  = 0.334 ± 0.009: heavily compressed along the *Z*_GSE_ axis. Figure 9c shows the best‐fit eccentricity of the ellipse as a function of the IMF clock angle range. Each point represents the fitted eccentricity of the IBEX MP crossings with corresponding IMF clock angles between |$\beta_{\text{min}}$| < |β| < |$\beta_{\text{max}}$|, listed on the *x*‐axis of figure. As the IMF clock angle range decreases, the eccentricity increases both gradually for some ranges and rapidly for others, peaking at e  = 0.435 ± 0.004 for the range 83° < |β| < 97°. The data points stop when not enough crossings are available to perform the fit. This trend indicates that during periods when |β| ∼ 90°, the magnetotail along the *Z*_GSE_ axis is compressed and thus prevailing the relation between the squeezing of the MP and the IMF clock angle.

## 6 Conclusions

Regions with high SW deflection, such as the magnetosheath and CME shock fronts, are observed in the IBEX‐Hi ENA imager as a large, anisotropic background signal, with a characteristic Gaussian dependence on IBEX spin angle and a maximum at the spin angle corresponding to the expected deflection that would cause the background in the IBEX‐Hi imager. If the responses to a series of consecutive swaths have a high peak‐to‐variance ratio, angle width near 52.9°, and a reduced chi‐square near unity, it is flagged as a magnetosheath boundary crossing.

This method of finding magnetosheath crossings, when applied to the first 352 IBEX orbits, produces 613 crossings. After correcting each crossing for the Earth's motion around the Sun, SW incident angle, and upstream SW conditions, and associating each crossing with a specific boundary, we obtain 280 BS crossings ranging from *X*_GSE_ ∼ 11 R_e_ to *X*_GSE_ ∼ −36 R_e_ and 241 MP crossings from *X*_GSE_ ∼ 6 R_e_ to *X*_GSE_ ∼ −48 R_e_. Some of these abrupt boundary crossings upstream are attributed to the simultaneous detection of passing ICMEs.

Using the normalization technique from Formisano et al. (1971), the IBEX boundary crossings obtained here generally coincide with the shape of the MP and BS inferred from previously used BS and MP data sets listed in the OMNIWeb database and the MMS BS crossings. This analysis provides the first extended crossing tailward near 30 R_e_ and 50 R_e_ for the BS and MP, respectively, filling a gap in prior in situ measurements. This IBEX data set extends further tailward into previously unexplored regions of the magnetosheath, making this data set useful for future BS and MP models. With deflected SW incident onto the IBEX‐Hi imager, we have provided evidence of an indirect way to identify MP and BS crossings encountered by IBEX. This method provides extended crossings in the far tail, which could be used to further refine and constrain MP and BS models. Furthermore, we have shown that IBEX inevitably enables identification of some magnetic boundary crossings (MP, BS, IP shocks) in its highly elliptical orbit.

With the IBEX data set's limited orbital inclination, we use IBEX's unique far tail crossings to provide observational evidence of magnetotail compression along the *Z*_GSE_ axis during periods when the IMF is quasi‐parallel to the ecliptic plane. Future models of the MP should account for the additional dependence on the IMF clock angle, β. Further observations of high |*Z*_GSE_| MP boundary crossings may also enable quantifying the relation between the IMF *B*_y_ strength and the degree of magnetotail compression.

## Data Availability

OMNI data were obtained from the GSFC/SPDF OMNIWeb interface at http://omniweb.gsfc.nasa.gov, which are derived from multi‐spacecraft SW plasma and magnetic field observations.
